# Model-based optimization and scale-up of multi-feed simultaneous saccharification and co-fermentation of steam pre-treated lignocellulose enables high gravity ethanol production

**DOI:** 10.1186/s13068-016-0500-7

**Published:** 2016-04-18

**Authors:** Ruifei Wang, Pornkamol Unrean, Carl Johan Franzén

**Affiliations:** Division of Industrial Biotechnology, Department of Biology and Biological Engineering, Chalmers University of Technology, Gothenburg, Sweden; National Center for Genetic Engineering and Biotechnology (BIOTEC), Pathum Thani, Thailand

**Keywords:** Biofuels, Fermentation technology, Agricultural residues, Bioprocessing, Enzymatic hydrolysis, High gravity, Demo-scale simultaneous saccharification and fermentation, Mathematical modelling, Fed-batch SSF

## Abstract

**Background:**

High content of water-insoluble solids (WIS) is required for simultaneous saccharification and co-fermentation (SSCF) operations to reach the high ethanol concentrations that meet the techno-economic requirements of industrial-scale production. The fundamental challenges of such processes are related to the high viscosity and inhibitor contents of the medium. Poor mass transfer and inhibition of the yeast lead to decreased ethanol yield, titre and productivity. In the present work, high-solid SSCF of pre-treated wheat straw was carried out by multi-feed SSCF which is a fed-batch process with additions of substrate, enzymes and cells, integrated with yeast propagation and adaptation on the pre-treatment liquor. The combined feeding strategies were systematically compared and optimized using experiments and simulations.

**Results:**

For high-solid SSCF process of SO_2_-catalyzed steam pre-treated wheat straw, the boosted solubilisation of WIS achieved by having all enzyme loaded at the beginning of the process is crucial for increased rates of both enzymatic hydrolysis and SSCF. A kinetic model was adapted to simulate the release of sugars during separate hydrolysis as well as during SSCF. Feeding of solid substrate to reach the instantaneous WIS content of 13 % (w/w) was carried out when 60 % of the cellulose was hydrolysed, according to simulation results. With this approach, accumulated WIS additions reached more than 20 % (w/w) without encountering mixing problems in a standard bioreactor. Feeding fresh cells to the SSCF reactor maintained the fermentation activity, which otherwise ceased when the ethanol concentration reached 40–45 g L^−1^. In lab scale, the optimized multi-feed SSCF produced 57 g L^−1^ ethanol in 72 h. The process was reproducible and resulted in 52 g L^−1^ ethanol in 10 m^3^ scale at the SP Biorefinery Demo Plant.

**Conclusions:**

SSCF of WIS content up to 22 % (w/w) is reproducible and scalable with the multi-feed SSCF configuration and model-aided process design. For simultaneous saccharification and fermentation, the overall efficiency relies on balanced rates of substrate feeding and conversion. Multi-feed SSCF provides the possibilities to balance interdependent rates by systematic optimization of the feeding strategies. The optimization routine presented in this work can easily be adapted for optimization of other lignocellulose-based fermentation systems.

**Electronic supplementary material:**

The online version of this article (doi:10.1186/s13068-016-0500-7) contains supplementary material, which is available to authorized users.

## Background

Agricultural residues such as wheat straw are attractive raw materials for fuel ethanol production since they may allow high resource efficiency while avoiding the competition for crops between food and fuel production. A promising process configuration for ethanol production from lignocellulosic feedstock is simultaneous saccharification and fermentation (SSF). In SSF, the enzymatic hydrolysis of the pre-treated biomass occurs simultaneously with the fermentation. SSF offers several advantages compared to separate hydrolysis and fermentation, including reduced end-product inhibition of hydrolytic enzymes caused by accumulated sugars and reduced operating cost because of the lower number of reactors needed. It has been estimated that the capital investment can be reduced by more than 20 % with SSF compared to separate hydrolysis and fermentation processes [1]. SSF also favours co-fermentation (SSCF) of glucose and xylose by recombinant *Saccharomycescerevisiae*, because the concentration of glucose can be kept low due to balanced rates of release and consumption by hydrolysis and fermentation [2].

Efficient and economical production of lignocellulosic ethanol requires high ethanol titres, since separation and rectification of the ethanol accounts for the major energy demand, in some cases more than 80 % [3]. The distillation cost can be reduced by operating SSF and SSCF at high content of water-insoluble solids (WIS), which means high sugar input and potentially high ethanol titre. Techno-economic models have predicted that increasing the insoluble solids content from 7 to 15 % (w/w) in a SSF process could reduce the energy demand by half at the same ethanol yield [3]. However, operation of SSF/SSCF at high solid concentration presents challenges such as high viscosity, resulting in low efficiency in mass and heat transfer and high power consumption for mixing. In addition, high substrate content also gives high concentrations of inhibitors, which affect the ethanol yield, titre and productivity negatively [4–7].

Using high enzyme dosage could accelerate saccharification and reduce viscosity quickly for high-WIS SSF/SSCF. However, the environmental impact and process economy of lignocellulosic ethanol are significantly affected by the enzyme usage [3, 8]. A fed-batch approach is preferred in this context since it allows relatively high enzyme to substrate ratios throughout the process by gradual addition of substrate. This has enabled substrate loadings up to 20 % (w/w) WIS in SSF and SSCF, and given relatively high final titres of ethanol up to 40 g L^−1^ with intermediate levels of enzymes [9, 10].

The smooth operation of fed-batch SSCF relies on balanced rates of substrate loading, hydrolysis and fermentation. Even though such balances are crucial for efficient mixing and productivity, they are difficult to achieve since every single rate in the SSCF context is interconnected with other rates via shared compounds. The full outcome of changes in one part of the process cannot be easily foreseen from intuition, nor easily derived from previous experience. Various strategies for fed-batch operation have been proposed, including controlled feeds of substrate, enzymes and cells [2, 9–13]. Yet, no systematic optimization of multiple feeding strategies for fed-batch SSCF has been reported, nor have they been tested in large-scale trials.

In this study, we carefully assessed the effects of substrate, enzyme and cell feeds on SSCF of SO_2_-catalyzed steam pre-treated wheat straw. We designed fed-batch profiles for high solid content processes by balancing the addition and hydrolysis of solid substrates via mathematical modelling and a control loop for determining the solid feeds. With such a model-driven approach, which predicted the dynamic outcomes of the feeds, we established balanced fed-batch SSCF by developing suitable feeding strategies regarding enzymes, substrates and cells. The process, termed multi-feed SSCF as it involved multiple feed streams, was scaled up to 10 m^3^ scale.

The objective was to improve ethanol production from pre-treated wheat straw by increasing the cumulative loading of water-insoluble solids (WIS), decreasing the enzyme usage and establishing procedures for yeast propagation with maximal use of pre-treatment liquor. The systematic optimization of multi-feed SSCF illustrated the important factors contributing to high ethanol titres. The optimization routine may be transferred to other lignocellulose-based processes.

## Results and discussion

### Overall scheme of multi-feed SSCF and strategy for optimization

**Fig. 1:**
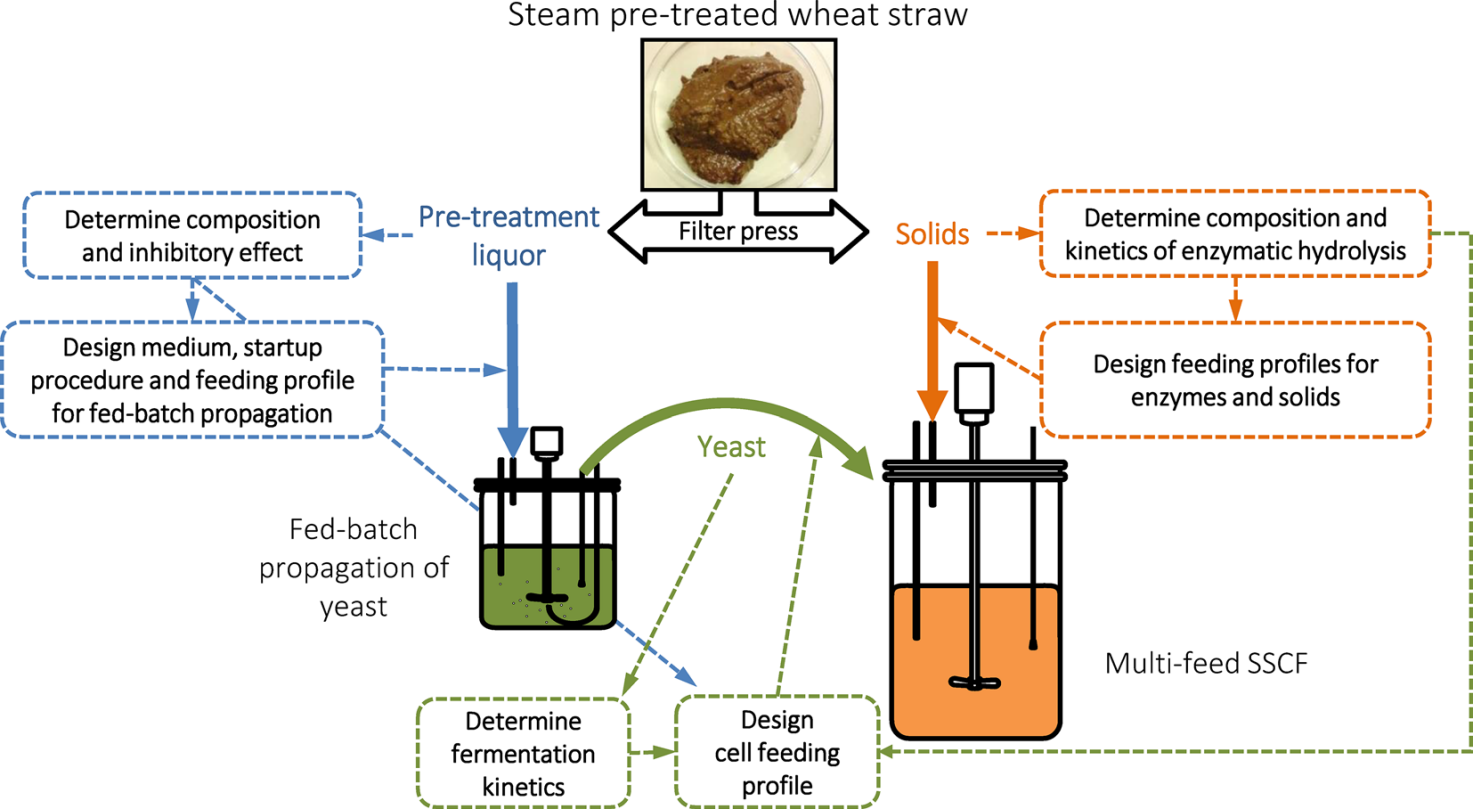
Scheme of multi-feed SSCF and process optimization targets. Pre-treated wheat straw from SP Biorefinery Demo Plant (BDP) contained WIS about 15–20 % (w/w). Separating solid from liquid fraction of the slurry by centrifugation (in lab) or filter press (in the plant) gave a solid fraction with about 40 % (w/w) WIS content. The multi-feed SSCF used solid fraction as substrate and allowed operation above 20 % (w/w) WIS. The liquid fraction was used for yeast propagation

The underlying hypothesis of this work was that efficient SSCF depends on balanced rates of the major reactions occurring within an SSCF, and that such balance can be achieved by controlling the rates of multiple feeds. The systematic optimization included three dimensions: feeding or not feeding enzymes; detailed solid feed profile and feeding or not feeding cells (Fig. 1). For enzymes and cells, comparison experiments were carried out and choices were made based on the results. For solids, feeding profiles were designed based on simulated rates of hydrolysis and fermentation, and the precondition that the broth viscosity had to be maintained low enough to enable good mixing.

### Yeast propagation in pre-treatment liquor is a compromise between high cell yield and high fermentation capacity

Cells adapted to the pre-treatment liquor during propagation have shown improved performance in SSF/SSCF [14–16]. Here, the objective was to optimize the addition of pre-treatment liquor to the medium and, hence, increase the adaptation pressure during cell propagation while still having rapid cell growth and high cell yield. The liquid fraction of pre-treated wheat straw contains oligomeric and monomeric sugars, and inhibitors that are formed during pre-treatment. The pre-treatment liquor can partly replace sugars and fresh water for cell production. However, it has been shown that yeast growth commences only after depletion of furfural and hydroxymethylfurfural [14, 17]. Therefore, higher level of pre-treatment liquor would lead to longer lag phase. In an industrial context, such lag phases must be avoided. Thus, the operation parameters for cell propagation are a compromise between yield, productivity and adaptation to the inhibitors.

**Fig. 2:**
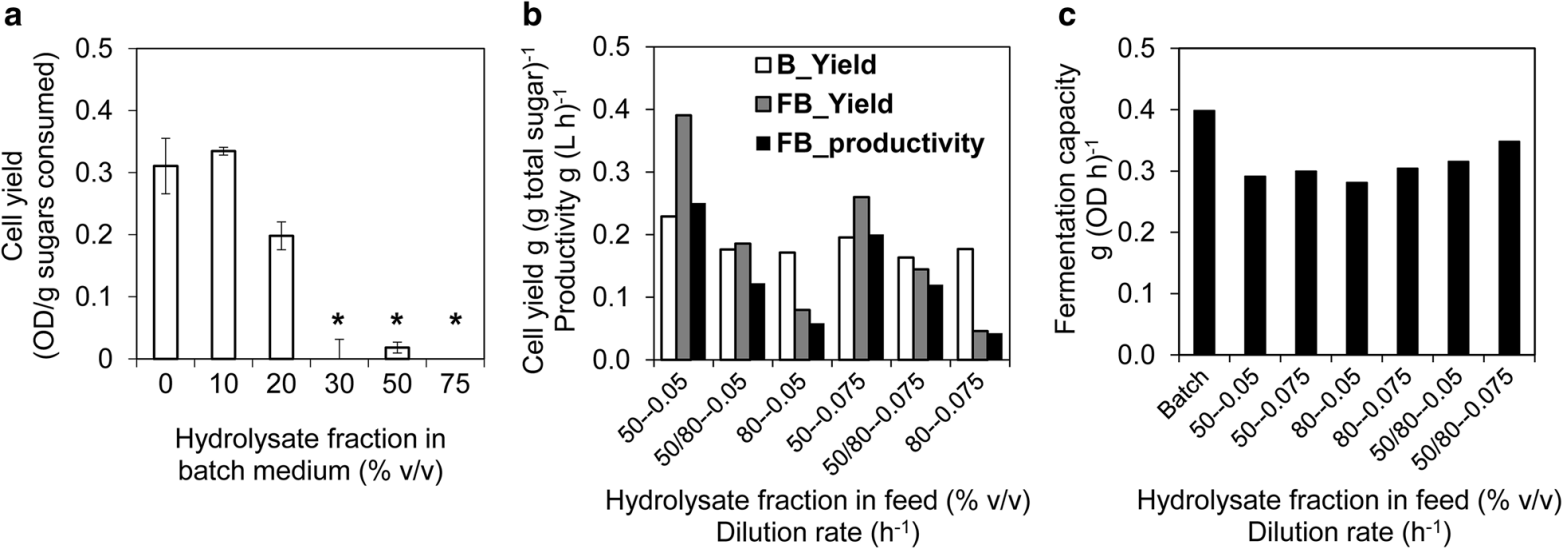
Optimization of seed cultivation in wheat straw pre-treatment liquor medium. **a** Effect of pre-treatment liquor concentration (% v/v) on cell yield after 24 h shake flask batch cultivation. The *asterisks* indicate that yeast growth was not sufficient to calculate the yield. *Error bars* indicate standard deviation of duplicate experiments. **b** Effects of the feed rate and the pre-treatment liquor concentration (% v/v) in feed medium on cell growth during fed-batch cultivation. Fed batch was started after a 24-h batch phase in 20 % (v/v) pre-treatment liquor medium. B_Yield, cell yields over the batch phase. FB_Yield, overall cell yield over the fed-batch phase, FB_Productivity, average cell productivity over the fed-batch phase. **c** Fermentation capacity of cells after batch and fed-batch cultivation. The conditions for seed cultivation in the subsequent multi-feed experiments were 20 % (v/v) pre-treatment liquor batch cultivation, followed by 50 % (v/v) pre-treatment liquor fed-batch cultivation at feed rate of 0.05 h^−1^

The cell propagation consisted of batch and fed-batch phases, and we optimized the medium composition for each phase (Fig. 2). In the batch phase, cell growth in 10 % (v/v) pre-treatment liquor medium was very similar to growth in the pre-treatment liquor-free medium, suggesting that at 10 % (v/v), this particular pre-treatment liquor was not toxic enough to affect cell growth. In 20 % (v/v) pre-treatment liquor medium, however, yeast cells grew only after a 12-h lag phase and the cell yield after 24 h was approximately 40 % lower than the yield obtained in 10 % (v/v) medium. In medium containing 30 % (v/v) pre-treatment liquor or more, no growth was observed within 24 h. Therefore, among the tested pre-treatment liquor contents in batch media, 20 % (v/v) was chosen as it imposed a significant pressure on cells for adaptation, while allowing sufficient cell growth in 1 day (Fig. 2a).

After the 24-h batch phase, yeast propagation was continued with fed-batch cultivation. High contents (50 and 80 %, v/v) of pre-treatment liquor were used in the feed medium and fed at dilution rates of 0.05 and 0.075 h^−1^. A higher fraction of pre-treatment liquor in the feed or a higher dilution rate may be positive for cell production and adaptation. However, it may also induce overflow metabolism and more sugar consumed for in situ detoxification, either of which reduces the biomass yield on sugars. Decreased cell yield and productivity were indeed observed with increasing fraction of pre-treatment liquor and increasing dilution rate (Fig. 2b). At the end of the fed-batch, residual ethanol was found in all cases except for the 50 % (v/v) pre-treatment liquor medium fed at 0.05 h^−1^. Furfural accumulation occurred at approximately 0.3 g L^−1^ when 80 % (v/v) pre-treatment liquor medium was used throughout the fed-batch phase. Therefore, 50 % (v/v) pre-treatment liquor in the feed and a dilution rate of 0.05 h^−1^ were chosen as they gave the highest overall cell yield and cell production rate, 0.39 g g^−1^ and 0.25 g (L h)^−1^, respectively, in the fed-batch (Fig. 2b).

The quality of yeast cells is crucial for fermentation processes. To evaluate cell quality, we determined the fermentation capacity in the presence of inhibitors (Fig. 2c). Cells prepared in the different fed-batch processes all had similar fermentation capacity, suggesting that the product quality did not depend on the feed rate and pre-treatment liquor concentration in the fed-batch phase. However, cells from batch cultivation showed higher ethanol productivity compared to those from fed-batch cultures, which had 70 % capacity of batch-cultured cells (Fig. 2c). This was likely because cells grew at higher specific growth rate in the respiro-fermentative batch phase, compared to the fed-batch phase [18]. The lower capacity might also be a result of reduced concentrations of inhibitors in the fed-batch culture due to in situ detoxification, which would lead to lower adaptive pressure in fed-batch propagation.

### Initial loading of all enzyme and model-based feeding of solids enabled rapid enzymatic hydrolysis at high solid loading

The efficiency of enzymatic hydrolysis relies on the properties of the pre-treated substrate, the activities of the enzyme cocktail and the process setup. We determined the enzyme dosage and the mode of enzyme addition based on the glucose release in batch and fed-batch hydrolysis experiments, respectively. We also developed substrate feed profiles using a control loop based on kinetic modelling. The results of hydrolysis experiments are summarized in Additional file 1: Table S1.

**Fig. 3:**
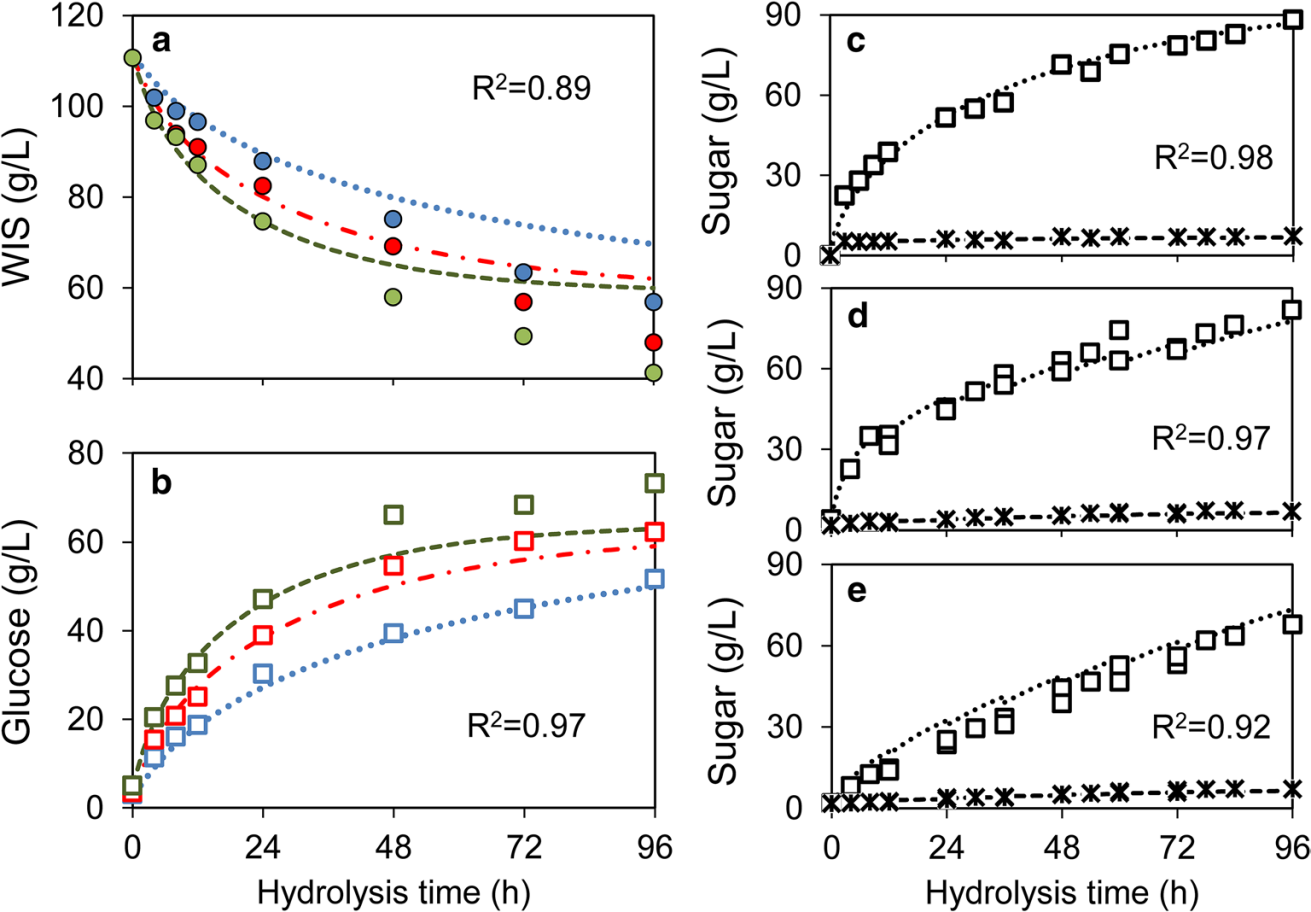
Fitting and validation of enzymatic hydrolysis model. **a** and **b** Concentrations of residual WIS (**a**) and of glucose (**b**) after fitting the hydrolysis model to batch experiments at 10 % (w/w) WIS using enzyme dosages of 5 (*blue*), 10 (*red*) and 15 (*green*) FPU (g WIS)^−1^. **c**–**e** Validation of the model was carried out by simulating the time course of glucose (*squares* and *dotted lines*) and xylose (*stars* and *dashed lines*) concentrations in a separate set of experiments using 15 % WIS and 10 FPU (g WIS)^−1^ in batch mode (**c**), fed-batch with all enzymes added initially (**d**) and fed-batch with enzymes added proportionally to substrate (**e**). Simulations are illustrated in *lines* and experiments in *symbols*. The coefficients of regression (*R* ^2^) are listed in each sub figure

10 % (w/w) WIS batch hydrolysis was carried out in bioreactors at the enzyme dosages 5, 10 and 15 FPU (g WIS)^−1^. The glucose yield after 96 h of hydrolysis was improved by 20 % when increasing the enzyme dosage from 5 to 10 FPU (g WIS)^−1^, and a further increase of 9 % was obtained with 15 FPU (g WIS)^−1^ compared to the 10 FPU case (Fig. 3a, b). Given these results, 10 FPU (g WIS)^−1^ enzyme dosage was selected for all following studies.

In fed-batch hydrolysis, loading all enzyme at time 0 clearly showed the advantage of boosting the initial hydrolytic rate and liquefaction of solid substrates, compared to gradual feeding of enzymes. Faster hydrolysis also resulted in more complete release of sugars from their polymeric forms. In experiments of 15 % WIS (w/w) fed-batch enzymatic hydrolysis, glucose yields at 96 h were 82 and 67 % of the theoretical yield with initial enzyme loading and split enzyme addition, respectively, at the fixed ratio of 10 FPU (g WIS)^−1^ (Fig. 3d, e; Additional file 1: Table S1). This indicates that mixing is of utmost importance for the initial as well as for sustained hydrolytic activities, and that unproductive adsorption to e.g. lignin and enzyme degradation are no major issues in the present case. Recently, the present view of irreversible and unproductive adsorption of hydrolases to lignin has indeed been challenged [19].

The key for successful fed-batch hydrolysis is to find a balance between the addition and degradation of solids. To predict the hydrolysis progress so that overfeeding could be avoided, we re-fitted a previously developed hydrolysis model (Eq. 1–4) [9] to steam pre-treated wheat straw.

1 $$\frac{{{\text{d}}E_{\text{ad}} }}{\text{dt}} = k_{\text{ad}} \cdot \left( {E_{\text{eq}} - E_{\text{ad}} } \right)^{2} ,$$

2 $$E_{\text{eq}} = E_{\text{load}} \cdot \frac{{S_{0} }}{S},$$

3 $$r_{\text{hydrolysis}} = k \cdot \frac{EC \cdot S}{{1 + G/K_{\text{G}} }} = k \cdot \frac{{E_{\text{ad}} C}}{{1 + G/K_{\text{G}} }},$$

4 $$r_{{X_{\text{n}} }} = r_{C} \cdot \gamma .$$

For nomenclature and optimized parameter values, see Table 1. The model was fitted by minimizing the residuals between the estimated and measured enzyme adsorption and concentrations of residual WIS, glucose and xylose during the 10 % (w/w) WIS batch hydrolysis (Fig. 3a, b). The fitted parameters (Table 1) were validated with a separate set of hydrolysis experiments, including 15 % (w/w) WIS batch hydrolysis and 15 % (w/w) WIS fed-batch hydrolysis experiments (Fig. 3c–e). Because they are fitted and validated on varied experimental datasets, kinetic models represent a set of collected information of the substrates, enzyme and their interactions. The model used here was originally developed for hydrolysis of birch [9], was adapted here for wheat straw, and has recently also been adapted for hydrolysis of corn stover [20], showing the wide validity and applicability of the model.

**Table 1:** Optimized parameters of the enzymatic hydrolysis and yeast fermentation

Parameters	Optimized value	95 % Confidence intervals	Unit	Description
$k_{\text{ad}}$	0.27	[0.03, 0.5]	g solid FPU^−1^ h^−1^	Adsorption rate constant
$k$	0.016	[0.009, 0.03]	g cellulose FPU^−1^ h^−1^	Hydrolysis rate constant
$K_{\text{G}}$	6.13	[2.7, 13.8]	g L^−1^	Inhibition constant of glucose in cellulose hydrolysis
$\gamma$	0.028	[0.01, 0.08]	–	Proportionality factor between xylan and cellulose degradation
$K_{\text{iEtOH}}$	16.6	Assumed^a^	g L^−1^	Inhibition constant of ethanol in cellulose hydrolysis
$q_{\text{G}}$	1.6	Assumed^a^	g g^−1^ h^−1^	Specific glucose uptake rate by yeast
$K$	0.01	Assumed^b^	g L^−1^	Saturation constant of glucose uptake
$Y_{\text{EtOH}}$	0.42	Assumed^b^	g g^−1^	Ethanol yield on glucose
$\alpha$	0.026	Assumed^b^	h^−1^	Pre-exponential factor of the ethanol-induced death rate coefficient
$\beta$	0.0037	Assumed^b^	L g^−1^	Exponential factor of the ethanol-induced death rate coefficient

^a^Modified from [9]

^b^Modified from [29]

**Fig. 4:**
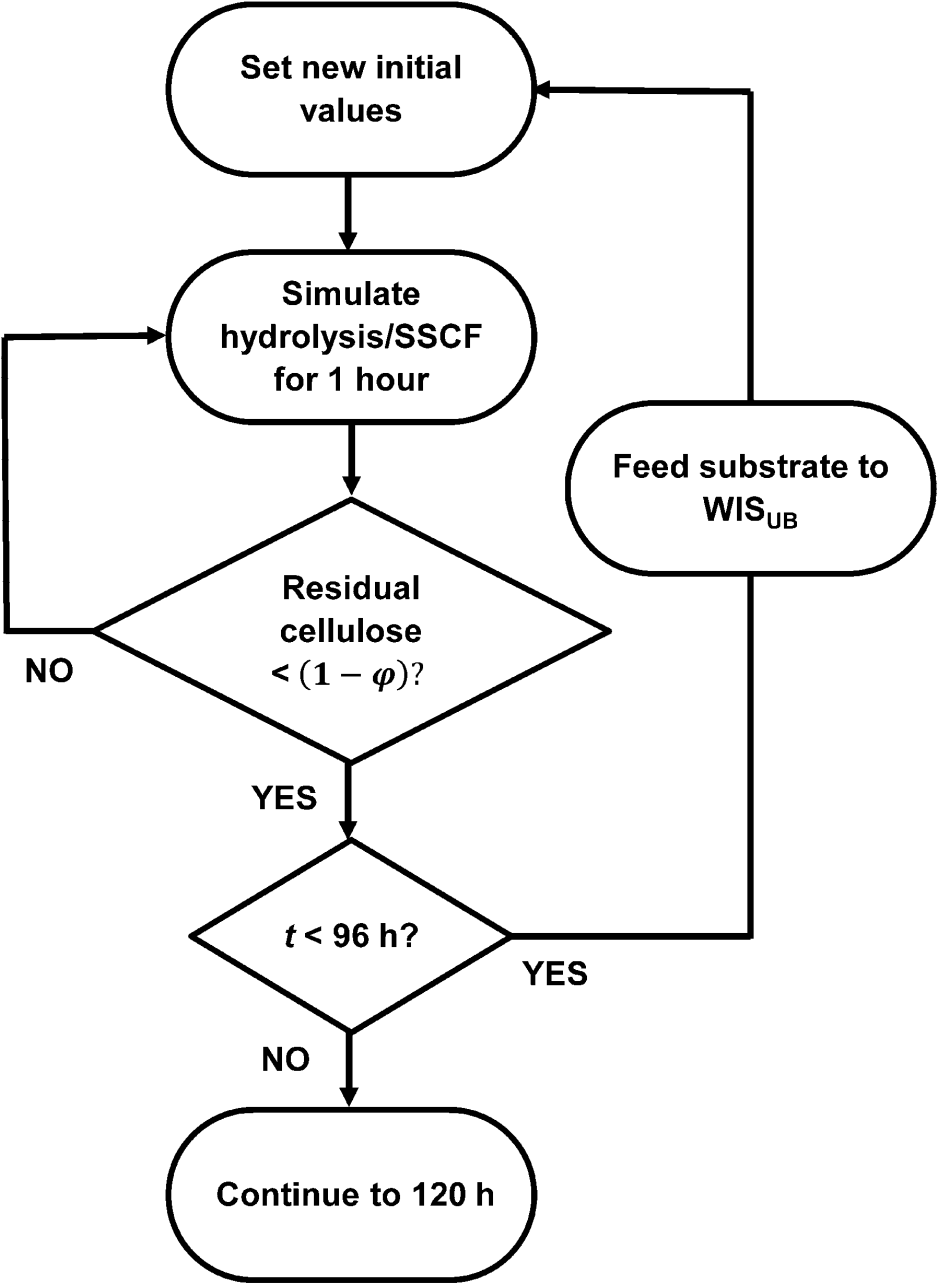
Schematic description of the model-based fed-batch design. After each 1 h simulation, the extent of cellulose degradation was checked. The process was simulated using $\varvec{\varphi }$ values of 30, 50, 60 and 80 % cellulose degradation. The upper boundary for the instantaneous concentration of WIS (WIS_UB_) in the bioreactor was determined to be 12–13 % (w/w)

The hydrolysis model was integrated into a model-based control loop which was developed for determining the times and amounts of solid feedings that would enable good mixing while promoting rapid turnover of cellulose (Fig. 4). Basically, a sequence of 1 h batch processes were carried out in silico. At the end of each batch simulation, the extent of WIS solubilisation (assumed equal to the cellulose degradation) was checked. If a certain fraction ($\varphi$) of the cellulose had been solubilized, a feeding event was triggered (Eq. 5). The amount of solids to be added was calculated based on the residual WIS and the mixing capacity of the reactors, expressed as an upper boundary for the WIS content in the reactor ${\text{WIS}}_{\text{UB}}$, empirically determined to be 12–13 % (w/w). Thus, at time *i*,

5 $$\begin{aligned}&if\,\, \frac{{C_{i - 1}^{'} - C_{i} }}{{C_{i - 1}^{'} }} > \varphi , \;\;{\text{then add solids corresponding to}} \\&\quad{\rm WIS}_{{{\text{add}},i}} = \frac{{ {\text{WIS}}_{\text{UB}} \cdot \left( {w_{i} + w_{\text{adds}} } \right) - {\text{WIS}}_{i} }}{{1 - {\text{WIS}}_{\text{UB}} /{\text{WIS}}_{\text{Solids}} }}, \end{aligned}$$

where $C_{i}$ is the residual amount of cellulose (g) at time *i*, $C_{i - 1}^{'}$ is the total amount of cellulose after the previous addition including the residual amount from the preceding time frame (g), $w_{i}$ is the total weight before the new addition (g), $w_{\text{adds}}$ is the increased weight by feedings other than solid substrates at time *i* (g), ${\text{WIS}}_{i}$ is the insoluble solids in the bioreactor at time *i* before the new addition (g) and ${\text{WIS}}_{\text{Solids}}$ is the WIS content in the solid fraction of the slurry (% w/w). Changes in volume and concentrations due to the feed event were immediately incorporated in the relevant variables and a new batch simulation was started from the updated initial values (Fig. 4).

The parameter $\varphi$ at the check point determines the feeding frequency and the time frame of the overall process. A feeding profile based on low cellulose conversion, e.g. $\varphi$ = 30 %, resulted in more frequent additions and consequently a possibility to reach higher cumulative WIS addition. However, the intense labour demands required by such a feeding plan could be impractical and may be unnecessary. With frequent additions, the apparent viscosity of the SSCF broth would be kept high and thus more power input for mixing would be required. In contrast, starting the feed of solids at higher degree of cellulose conversion would ensure sufficient mixing, but would prolong the process time needed to reach the same total WIS addition. After simulation and experimental tests, feeding upon 60 % cellulose degradation ($\varphi$ = 60 %) was chosen since it gave desirable WIS addition with reasonable work load. Fed-batch hydrolysis carried out according to model predictions using these parameters worked smoothly and no mixing problems were encountered.

In conclusion, adding all enzymes initially accelerates the liquefaction of the medium. The rapidly reduced viscosity in the early stages of the process resulted in better mixing throughout the whole process. Furthermore, feeding of solids according to the predicted extent of hydrolysis enabled a balance between process time and the overall substrate addition, while reducing the risk of overfeeding.

### Model-based feeding of solids and addition of yeast resulted in the most productive SSCF at high solid loading

In the SSCF set up, glucose and xylose released during hydrolysis are taken up by yeast cells and converted to ethanol. We investigated how the process performance was affected by feeding of not only solids, but also of enzymes and yeast cells. Experimental results are summarized in Additional file 2: Table S2.

#### Enzyme feeding in SSCF

Enzyme feeding has previously been shown to improve the overall performance of SSCF due to enhanced xylose utilization, by limiting the glucose release and maintaining a favourable xylose to glucose ratio during the process [2, 10]. However, according to our results at high solid loading of wheat straw, the appropriate enzyme feeding mode appears to be adding all enzymes at the start in order to accelerate liquefaction of the medium. With all enzymes added initially in the SSCF, ethanol production was boosted in the early phase and final concentrations were higher compared to SSCF with enzyme feed; for example, the ethanol yield on total sugars at 96 h increased from 77.8 to 81.5 % of the theoretical yield in 15 % (w/w) WIS fed-batch SSCF (Additional file 2: Table S2).

To enable its use in the SSCF context, the hydrolysis model was extended to include ethanol inhibition of enzymatic hydrolysis (Eq. 6), glucose consumption ($r_{{{\text{G}} , {\text{cons}}}}$, Eq. 7), ethanol production ($r_{\text{EtOH}}$, Eq. 8) and ethanol-induced cell death ($r_{d}$, Eq. 9) to represent the main reactions involved in an SSCF which could affect the hydrolysis process (for nomenclature and parameter values, see Table 1).

6 $$r_{{{\text{G}} , {\text{prod}}}} = \frac{{r_{\text{hydrolysis}} }}{{1 + {\text{EtOH}}/K_{{i{\text{EtOH}}}} }},$$

7 $$r_{{{\text{G}} , {\text{cons}}}} = \frac{{q_{G} \cdot G}}{K + G} \cdot X,$$

8 $$r_{\text{EtOH}} = r_{{{\text{G}} , {\text{cons}}}} \cdot Y_{\text{EtOH}} ,$$

9 $$r_{\text{d}} = \alpha \cdot e^{{\beta \cdot {\text{EtOH}}}} \cdot X.$$

**Fig. 5:**
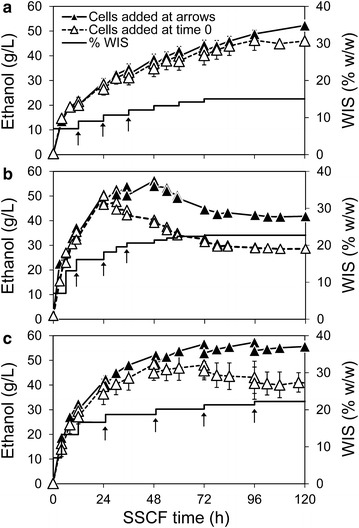
Effects of cell feeding on SSCF performance. Feeding cells improved the overall performance of fed-batch SSCF process (*filled triangles*) compared to loading all cells initially at 0 h (*open triangles*), in 15 % (w/w) final accumulated WIS addition (**a**), 22 % (w/w) final WIS with unoptimized addition of solids and insufficient mixing after 24 h (**b**), and 22 % (w/w) final WIS with optimized addition of solids and efficient mixing (**c**). At high accumulated WIS concentration, stress induced by inhibitors and ethanol is more severe than at low WIS contents. Consequently, the benefit of feeding cells instead of adding them all initially was clearer. The *arrows* indicate addition of yeast cells in the experiments represented with *filled triangles*. The *error bars* indicate standard deviation of duplicate experiments

**Fig. 6:**
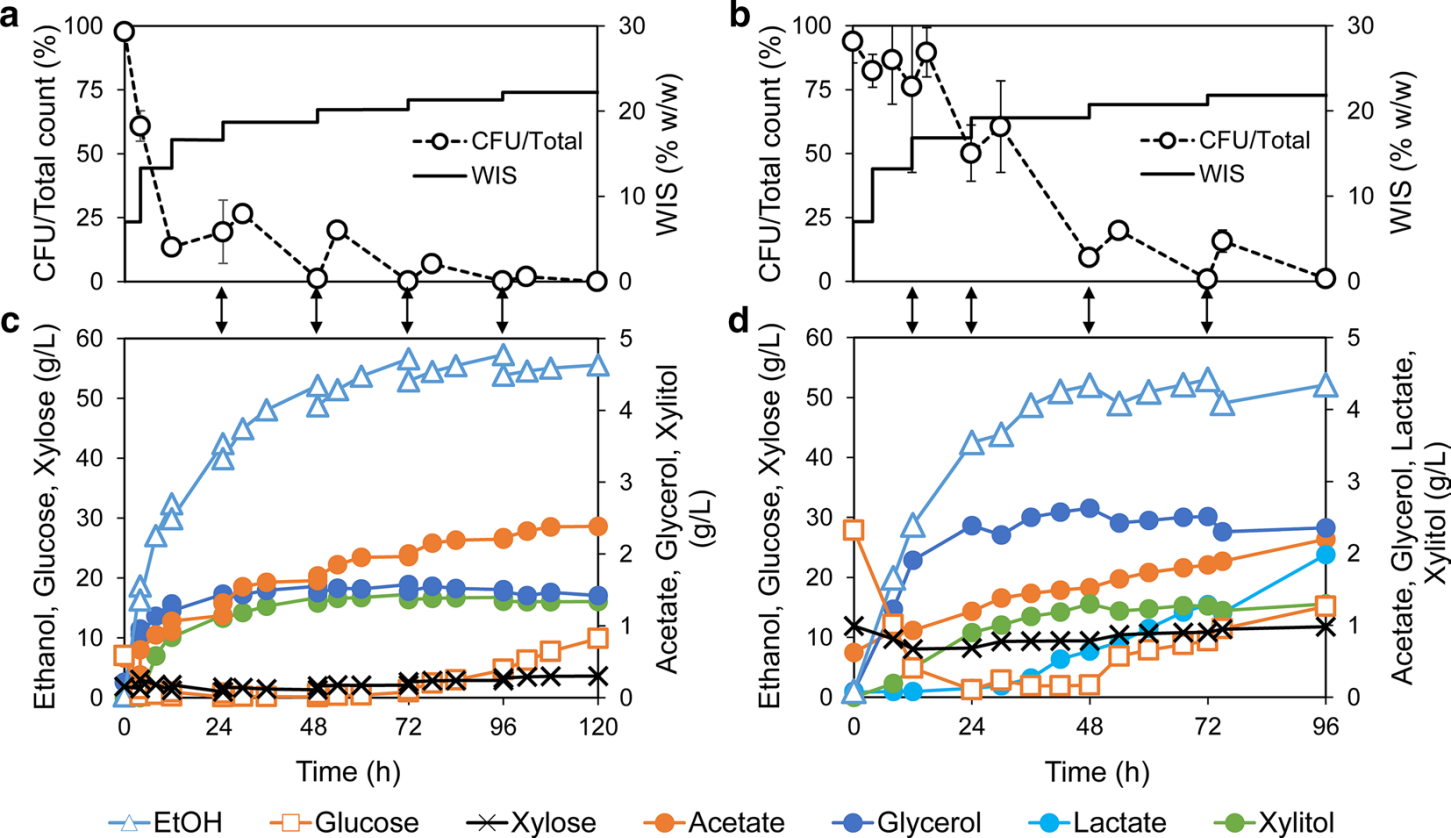
Comparison of lab and demo-scale SSCF. Time courses of cell viability, cumulative WIS addition, concentrations of sugars, ethanol and major by-products of multi-feed SSCF in lab scale (**a**, **c**) and in demo plant (**b**, **d**). The changes in WIS content indicate addition of solids, and the *arrows* between the panels indicate addition of yeast cells. Concentrations in C and D are averages from two biological replicates. Most data points showed deviation below 5 % between duplicate experiments. *Error bars* in **a** and **b** are standard deviations in duplicate experiments. Furfural and 5-hydroxymethyl furfural concentrations were below 0.5 and 0.2 g L^−1^ in the lab case, and below 0.1 and 0.05 g L^−1^ in the demo case

The resulting model was integrated into the process design loop to determine the amounts and times for solid feeds (Fig. 4). Since inhibition of enzyme by glucose was weaker, the hydrolysis was faster and solid feeds were scheduled earlier in the SSCF compared to those in the separate hydrolysis process. SSCF experiments with substrate feeding based on the model prediction were carried out in both laboratory and demonstration scales. 22 % (w/w) overall WIS addition was achieved without mixing problem throughout the process (Figs. 5, 6).

#### Yeast cell feeding in SSCF

A sufficient inoculum size for an SSCF process implies that the amount of cells added is enough to consume glucose at the rate of release, given the inhibitor concentrations in the medium. Selection of the appropriate inoculum size was carried out in shake flask batch SSCF with 20 % (w/w) WIS and 10 FPU (g WIS)^−1^ enzyme with 2 h pre-hydrolysis to enable mixing. Batch SSCF is more challenging for cells than fed-batch because all inhibitors are present at the beginning of the batch. After 2 h pre-hydrolysis, inocula equivalent to 0.01, 0.02 and 0.04 g cell dry weight (DW) (g WIS)^−1^ were added. Increased ethanol production by 12 % (37.7–42.2 g L^−1^) after 24 h incubation was obtained when the cell loading was increased from 0.01 to 0.02. When the inoculum size was further increased to 0.04 g (g WIS)^−1^, no improvement in ethanol production was observed. The results suggested that at such WIS and enzyme dosage, 0.02 g (g WIS)^−1^ cell loading was sufficient to consume available sugars while handling high level of inhibitors. Thus, 0.02 g DW (g WIS)^−1^ were used in all SSCF experiments.

The amount of viable yeast cells, measured by colony forming assay, has been found to correlate with the ethanol production during SSF/SSCF of pre-treated birch, spruce and wheat straw, and the viability, estimated as colony forming units (CFU) per total cell count, often decreases during SS(C)F at high gravity [9, 10, 15, 21]. Feeding cells, in other words, split inoculation, has been shown to effectively improve the viability of the population as well as the ethanol production in 20 % (w/w) WIS SS(C)F on birch, spruce and corn stover [9, 10, 20]. In this work, the effects of cell feeding on SSCF were examined under varied solid feeding schemes. At the intermediate WIS addition of 15 % (w/w), feeding cells showed small effects on the process but slightly increased the final ethanol titre (Fig. 5a), likely due to that yeast metabolism was not completely eliminated by the inhibitors and the produced ethanol at this WIS level. In cases of an overfed 22 % (w/w) WIS SSCF, where proper mixing could not be maintained, cell feeding to some extent salvaged the process and resulted in continued ethanol production (Fig. 5b). In the model-based 22 % (w/w) WIS SSCF, where mixing was maintained throughout the process, cell feeding also led to maintained fermentation and final ethanol concentrations of more than 55 g L^−1^. In contrast, fermentation ceased above ethanol concentrations of 45–50 g L^−1^ in SSCF with initial cell inoculation (Fig. 5c).

Thus, our results explicitly pointed out that it is more beneficial to feed cells at the later stage of SSCF, when the ethanol concentration is high and viability is low. In this case, feeding of continuously propagated cells maintained the fermentation activity and further improved ethanol yields. Feeding cells at lower ethanol level (roughly below 45 g L^−1^) made little difference in final ethanol titres (Fig. 5). If higher ethanol titre is the target and thus continuation of fermentation is required, addition of cells is a simple and effective way to maintain sufficient cell viability.

Taken together, by extending fed-batch SSCF to include feeds of not only solid substrate, but also cells and in some cases enzymes, it is possible to achieve balanced rates of substrate feeding and hydrolysis, sugar release and uptake, ethanol production and cell viability. The model-based process design presented here provides a versatile working platform for high gravity lignocellulose-based processes.

### Scale-up of lab-optimized Multi-feed SSCF process to demo scale gave similar results as in lab

The lab-optimized multi-feed SSCF and yeast cultivation processes were carried out in 10 m^3^ scale at the SP Biorefinery Demo Plant in Örnsköldsvik, Sweden, after adaptation to the plant’s capabilities (see operating conditions in Table 2). A new batch of pre-treated raw material had to be produced, and despite identical operating set points the new pre-treated slurry differed from the one used in the lab experiments in terms of sugar composition and inhibitor concentrations (Table 3).

**Table 2:** Comparison of the lab-scale ‘best’ performing SSCF and accordingly designed demo-scale SSCF

	Lab	Demo plant
Total weight	1252 g	5481/5583 kg^a^
WIS loading (% w/w)	22.2	21.9/21.5^a^
Pre-treatment liquor	0	178/196 kg^a^
Operating time	120 h	96 h
Cells added at	0, 24, 48,72 and 96 h	0, 12, 24, 48 and 72 h
Solids added at	0, 4, 12, 24, 48, 72 and 96 h	0, 4, 12, 24, 48 and 72 h

^**a**^The actual operation data for duplicate experiments

**Table 3:** Compositions of the two batches of pre-treated wheat straw used in lab and demo plant

Contents in solid phase (% g (g WIS)^−1^)			Contents in liquid phase (g L^−1^)		
	Lab	Demo		Lab	Demo
Glucan	47.9	42.4	Glucose	6.8	2.4
Xylan	2.3	2.6	Xylose	12.8	18.3
Mannan	0.2	0.2	Mannose	0.4	0.4
Galactan	0.04	0.0	Galactose	1.0	0.8
Arabinan	0.1	0.08	Arabinose	2.0	2.8
			Acetic acid	3.8	3.2
			Furfural	4.0	0.8
			5-Hydroxymethylfurfural	1.4	0.4

The time courses of cell viability, cumulative WIS addition, major sugars, ethanol and by-products are compared with lab-scale results in Fig. 6. Despite the differences in substrate and scale, the multi-feed SSCF performed similarly overall in the two cases. In the first 48 h, the cell viability was higher in the demo-scale experiments because of the lower inhibitor concentrations in the pre-treatment liquor. Due to slower cell separation, the initial cell addition at 0 h was lower in the demo than in the lab experiments. Fresh cells were instead added already after 12 h, which also boosted the viability. In lab experiments, the ethanol concentration reached 57.3 g L^−1^ and the yield on total sugar was 66 % of the theoretical at 96 h (Fig. 6a, c). No mixing issue was observed throughout the process. In the demo trials, the ethanol concentration peaked at 53.0 g L^−1^ at 72 h, and the final yield on total sugar at 96 h was 54 % of the theoretical (Fig. 6b, d). The results were quite reproducible in the demo scale, with less than 5 % deviation between the two repeated experiments.

Large-scale operation is complicated and unexpected issues can happen. Therefore a process going for large scale must be robust and resistant to mistakes and deviations in operations. In fuel ethanol production, contamination is difficult to eliminate since the process is usually carried out under non-sterile conditions [22]. The fed-batch strategy used in both yeast propagation and SSCF processes kept the sugar level low and therefore made it more difficult for bacteria to take over. Increasing the use of inhibitory pre-treatment liquor during both yeast propagation and SSCF could further repress bacterial growth, however, this must be weighed against the potential negative effects on the ethanol fermentation.

In this context, multi-feed SSCF is a flexible process which balances substrate input, saccharification and fermentation, by allowing adjustment of the feed rates according to the circumstances during a run. In the demo trials, we noticed that feeding solids to the SSCF and separation of yeast from the propagation medium were two time-consuming steps. For feasible application of the multi-feed strategy in large scale, use of automated and continuous solid feeding, and more powerful cell separation or use of flocculent yeast to facilitate separation of cells from the propagation medium should be considered [23]. Despite these issues, the process was quite robust and lab-scale results were reproduced in the demo scale.

### Development of multi-feed SSCF on varied raw materials showed the important factors for high ethanol titre

The strategy of combining substrate, enzyme and yeast feed in SS(C)F has been developed and tested on pre-treated birch [9] and spruce [10], and was further developed on wheat straw in this work. On birch, multi-feed SSCF did not give high ethanol titres, but clearly improved the reproducibility [9]. One clear reason for the low product concentration was that the birch pre-treatment liquor contained quite high inhibitor concentrations, like 18.3 g L^−1^ acetic acid and 3.7 g L^−1^ furfural. Therefore, with increasing overall substrate loads, the ethanol titres actually decreased. On spruce, feeding of enzymes actually increased the ethanol titre when combined with feeds of yeast and solids. By using 50 % (v/v) spruce pre-treatment liquor in the cell propagation and a very frequent manual substrate addition (every 4 or 8 h), relatively high ethanol titres of 40 g L^−1^ were achieved in a 20 % (w/w) WIS process [10]. In the current work on steam pre-treated wheat straw, loading all enzyme at beginning of the process clearly boosted both the enzymatic hydrolysis and the SSCF, and high final concentrations of ethanol were achieved. The high level of ethanol became challenging for the yeast when combined with other inhibitors. Therefore, the effect of the yeast feed was to maintain the fermentation at the later stages of the SSCF rather than boosting it in the early stages.

The different results obtained in these studies are in part due to the different nature of the raw materials, i.e. hardwood, softwood and agricultural residues, respectively. The differences are also an outcome of a maturation process of the multi-feed concept. In this work, planning of the substrate feeds according to model prediction reduced the feeding frequency. This required less labour in manual operations, but still resulted in higher overall WIS within a similar time frame. Moreover, in this study, the procedures for cell propagation were well established and integrated with the process. We used a mixture of molasses and pre-treatment liquor for providing sugars and adaptation pressure during cell propagation. The semi-continuous cultivation delivered cells with stable viability and fermentation capacity. The multi-feed process was practical for scaling-up and delivered similar results in 10 m^3^ scale as in the lab. The final yield on total sugars must be further improved, which will lead to even higher final ethanol titres. We also found that the ethanol concentration peaked before the last substrate addition. Therefore an alternative process setup would be to skip the last two substrate additions. In conclusion, the multi-feed SSCF process presented here represents a complete routine for establishing a high gravity, i.e. high solids content, production workflow from pre-treated lignocellulose to value-added products (Fig. 1).

## Conclusions

We have demonstrated a dynamic approach to process development, using kinetic modelling to predict the feeding of solids and the outcomes of such feedings. With the model-based feeding profiles, it is possible to reach a high-WIS content of 22 % (w/w) without mixing issues in a standard bioreactor, resulting in high ethanol concentrations (57.3 g L^−1^) from pre-treated wheat straw. For obtaining a successful multi-feed SSCF process, adding all enzymes at the beginning is a key factor for high titre and productivity. This is also beneficial for the economic and environmental impact of the process since the enzyme requirement can be reduced. Feeding yeast is effective for maintaining the viability and fermentation activity. The systematic optimization reported in this work represents a robust and reproducible routine for development of other lignocellulose-based process.

## Methods

### Raw material

Pre-treated wheat straw used throughout this study was provided by SP Biorefinery Demo Plant (Örnsköldsvik, Sweden). The wheat straw was steam pre-treated with 1 % (w/v) H_2_SO_4_. The biomass slurry after pre-treatment was separated by filtration into a water-insoluble solid (WIS) fraction and a liquid hydrolysate fraction, here called pre-treatment liquor. The solid fraction was used for hydrolysis and SSCF experiment, while the pre-treatment liquor was used for seed cultivation (Table 3). The sugar composition in the solid fraction was determined according to the NREL protocol TP-510-42618 [24]. The sugar and inhibitor composition of pre-treatment liquor was determined by IC and HPLC measurement, respectively. WIS content for the pre-treated biomass was determined by weighing an aliquot of the solid fraction, thorough washing with deionized water and weighing after 24 h drying at 105 °C. Enzyme activity in filter paper units (FPU) was determined according to the NREL protocol TP-510-42628 [25] with reduced reaction volume [26].

### Strain and media

The recombinant and evolutionary engineered strain of *S. cerevisiae* KE6-12.A [27] capable of fermenting glucose and xylose was used in all SSCF experiments. The strain was maintained as a frozen glycerol stock. Prior to use, the strain from frozen stock was streaked on a fresh YPD agar plate containing 10 g L^−1^ yeast extract, 20 g L^−1^ peptone, 20 g L^−1^ agar and 20 g L^−1^ glucose. The media used for all hydrolysis and SSCF experiments contained pre-treated wheat straw, 0.5 g L^−1^ (NH_4_)_2_HPO_4_ and 125 µL L^−1^ of Vitahop (BetaTech GmbH, Schwabach, Germany) to reduce the risk of contamination. The pH of the media was adjusted to 5.0 with 3 M NaOH. All enzyme hydrolysis, seed cultivation and SSCF experiments were carried out in 3.6 L Labfors bioreactors (INFORS HT, Bottmingen-Basel, Switzerland) unless otherwise stated. The bioreactors were autoclaved at 121 °C for 20 min prior to the experiments.

### Enzymatic hydrolysis

Cellic CTec II enzymes (Novozymes, Denmark) were used in this work. Batch and fed-batch hydrolysis were conducted with final accumulated WIS of 15–21 % (w/w, dry basis). For enzyme dosage optimization experiment, enzyme loading was varied at 5, 10 and 15 FPU (g WIS)^−1^. The hydrolysis conditions were temperature at 35 °C, pH controlled at 5 with 3 M NaOH and agitation rate at 400 rpm. The hydrolysis was initiated by adding enzyme preparations to the bioreactor. Fed-batch was carried out by feeding solid and/or enzyme according to the designed feed profiles. Samples for WIS, free enzyme and sugar analysis were taken every 6 or 12 h.

### Aerobic batch and fed-batch seed cultivation

Yeast cells used in SSCF were produced in aerobic batch followed by fed-batch culture using wheat straw pre-treatment liquor to allow cells to adapt to the used medium. Inoculum culture was grown in liquid YPD medium in a 250-mL Erlenmeyer flask at 30 °C at a shaker speed of 200 rpm for 24 h. The culture was initiated by inoculating the YPD culture at 10 % (v/v) to the batch. The composition of the batch medium was 10 % (v/v) molasses, pre-treatment liquor at various amounts as indicated below, 7.5 g L^−1^ (NH_4_)_2_SO_4_, 3.5 g L^−1^ KH_2_PO_4_, 0.7 g L^−1^ MgSO_4_∙7H_2_O, 2 mL L^−1^ trace metals and 1 mL L^−1^ vitamins. The trace metal and vitamin solutions were prepared according to [28]. Glucose and xylose were added to obtain initial concentrations of 20 g L^−1^. Pre-treatment liquor was added to the batch media at 0, 10, 20, 30, 50 and 75 % (v/v). The batch phase was carried out for 24 h followed by fed-batch cultivation. The fed-batch phase was commenced by adding feed media at the specified dilution rate. Two dilution rates of 0.05 and 0.075 h^−1^ were tested. Different feed media were tested with the following compositions: 2 L of 50 % (v/v) pre-treatment liquor, 2 L of 80 % (v/v) pre-treatment liquor and 1 L of 50 % (v/v) pre-treatment liquor followed by 1 L of 80 % (v/v) pre-treatment liquor. The feed media contained the same levels of molasses and salts as the batch media. Batch and fed-batch cultivations were performed at 30 °C and pH was maintained at 5 by addition of 3 M NaOH. Aeration was maintained at 1 L min^−1^ during batch and at 2 L min^−1^ during fed-batch. The stirrer speed was kept at 400 rpm during batch and at 800 rpm during fed-batch. Dissolved oxygen stayed above 20 % of air saturation throughout the cultivation. Cells were harvested by centrifugation at 5100 rpm for 5 min. The cell pellets were washed and re-suspended in 0.9 % (w/v) NaCl solution before being used for SSCF fermentation.

### SSCF in shake flasks

Batch shake flask SSCF was carried out in a 250 mL baffled Erlenmeyer flask containing pre-treated wheat straw at 20 % (w/w) WIS content. The pH was initially adjusted to 5 using 3 M NaOH. Before inoculation, 2 h pre-hydrolysis at 50 °C was performed to partially solubilize the solid and facilitate mixing. SSCF was initiated by addition of various amounts of yeast cells equivalent to 0.01, 0.02 and 0.04 g (g WIS)^−1^. The SSCF was conducted at 35 °C, with agitation rate at 180 rpm with no pH control during the cultivation. Samples were taken every 24 h for cell, sugars and ethanol measurement.

### SSCF in bioreactors

10 FPU (g WIS)^−1^ enzyme loading and yeast cell ratio of 0.02 g (g WIS)^−1^ were used in bioreactor SSCF experiments. Fed-batch SSCF experiments were started with initial WIS of 7 % (w/w) and were fed to the final WIS content, with co-feeding of enzymes and/or yeast cells. When enzyme feeding was applied, enzyme preparations equivalent to 10 FPU (g added WIS)^−1^ were added to the bioreactor at each feeding. In cases with cells feedings, cells equivalent to 0.02 g (g added WIS)^−1^ were added to the bioreactor at each feeding. To keep the working volume the same under different feeding schemes, water equal to the amount of enzyme or cell solution added was used for the experiment with no cell and/or enzyme feeding. Samples were collected and analysed for sugars, fermentation products, residual inhibitors and cell concentrations. The process was conducted at 35 °C, with agitation rate at 400 rpm and pH at 5 by addition of 3 M NaOH.

### Multi-feed SSCF in demo plant

At SP Biorefinery Demo Plant, the lab-optimized multi-feed SSCF and yeast cultivation were carried out in large reactors (10 m^3^). A new batch of wheat straw was pre-treated to obtain sufficient materials for the experiments (Table 3). The operating parameters for yeast cultivation and SSCF were basically the same as those optimized in the lab, but some modifications were made to fit the facilities in the plant. For yeast cultivation, a 300-L bioreactor was used to grow cells before the major cultivation in a 10-m^3^ bioreactor. The numbers of yeast cells and bacteria in all cultivation steps were regularly monitored to follow yeast growth and risk of contamination. A continuous centrifuge with working capacity of 300 L h^−1^ was used to prepare concentrated cell slurry for SSCF. Before the start of the SSCF, a gentle pre-hydrolysis was initiated to facilitate mixing. During the SSCF, 450–600 kg of pre-treated solids were added from the top of the bioreactor at every feeding point. It took several hours until all the solids were mixed with the SSCF broth after each addition.

### Analytical procedures

#### Cell concentration

Cell concentration was measured by cell dry weight during the aerobic seed cultivation. The samples were centrifuged at 5100 rpm for 5 min and the supernatants were discarded. The cell pellets were washed with deionized water, filtered through a pre-weighed 0.2 µm filter paper (PESU-membrane), dried at 105 °C for 24 h and subsequently weighed. During SSCF cultivation, the concentration of viable cells was measured by manual counting of colony forming units (CFU). CFU counts were obtained by plating 0.1 mL of sample on an YPD plate, after appropriate serial dilution. Total cell concentration was measured by counting cells under microscope using a counting chamber. Optical Density (OD) was measured by absorbance at 600 nm after appropriate dilution and zeroing using filtered liquid medium.

#### Analysis of sugars, fermentation products and inhibitors

Hydrolysis and fermentation samples were centrifuged at 5100 rpm for 5 min. The supernatant was collected and filtered using a 0.2-µm filter. Samples were stored at −20 °C prior to analysis. Concentrations of monomeric sugars (glucose, xylose, arabinose, galactose and mannose) were analysed on a high performance anion exchange chromatography system (ICS 3000, Dionex) with Dionex CarboPac PA1 guard and analytical columns (Thermo Scientific), and pulsed amperometric detection. Milli-Q water was used for sample elution at a flow rate of 1 ml/min, and 300 mM NaOH was added at a flow rate of 0.5 ml/min before the detector. The column was regenerated between sample injections using a mixed eluent consisting of 20 % Milli-Q water, 40 % 300 mM NaOH and 40 % 100 mM NaOH + 85 mM sodium acetate, followed by equilibration with Milli-Q water. Concentrations of fermentation products (glycerol, xylitol and ethanol) and inhibitors (acetic acid, 5-hydroxymethylfurfural and furfural) were determined on an UltiMate 3000 HPLC system with a Variable Wavelength absorbance detector set at 210 nm (Dionex) and an IR-101 refractive index detector (Shodex), using a Phenomenex Rezex ROA column. Samples were eluted at 80 °C using 0.8 ml/min 5 mM H_2_SO_4_. The concentrations were calculated from calibration curves for standard solutions.

#### Fermentation capacity test

1 mL cell sample was collected after the end of batch and fed-batch seed cultivation. The cell sample was washed once with deionized water before being inoculated into 5 ml culture medium containing 10 % (v/v) pre-treatment liquor, 7.5 g L^−1^ (NH4)_2_SO_4_, 3.5 g L^−1^ KH_2_PO_4_, 0.7 g L^−1^ MgSO_4_·7H_2_O. The culture was incubated at 30 °C with a shaking rate of 200 rpm for 2 h. Supernatant samples from the culture were collected for sugars and ethanol analysis.

#### Yield and rate calculations

Hydrolysis sugar yields were calculated by dividing the maximum amount of released sugar (g) by the total amount of polymeric sugars available in the water-insoluble solid (WIS) fraction of the pre-treated wheat straw. Ethanol yields were calculated by dividing the total amount of ethanol produced by the total amount of fermentable sugars available in the added liquids and solids. Cell yields were based on the total cell dry weight or OD produced per total sugar available or consumed, as indicated in the text. The average cell production rate was estimated based on the total cell dry weight produced per total cultivation time.

## Additional files

10.1186/s13068-016-0500-7 Contains experimental results from enzymatic hydrolysis experiments
10.1186/s13068-016-0500-7 Contains experimental results from multi-feed SSCF experiments

## Abbreviations

CFU: colony forming units
DW: dry weight
FPU: filter paper units
HPLC: high performance liquid chromatography
OD: optical density
SSCF: simultaneous saccharification and co-fermentation
SSF: simultaneous saccharification and fermentation
WIS: water-insoluble solids
YPD: yeast extract, peptone, dextrose medium
*k*_ad_: adsorption rate constant (g solid FPU^−1^ h^−1^)
*k*: hydrolysis rate constant (g cellulose FPU^−1^ h^−1^)
*K*: saturation constant of glucose uptake (g L^−1^)
*K*_G_: inhibition constant of glucose in cellulose hydrolysis (g L^−1^)
*K*_iEtOH_: inhibition constant of ethanol in cellulose hydrolysis (g L^−1^)
*q*_G_: specific glucose uptake rate by yeast (g g^−1^ h^−1^)
*Y*_EtOH_: ethanol yield on glucose (g g^−1^)
*α*: pre-exponential factor of the ethanol-induced death rate coefficient (h^−1^)
*β*: exponential factor of the ethanol-induced death rate coefficient (L g^−1^)
*γ*: proportionality factor between xylan and cellulose degradation (−)
*φ*: fraction of cellulose that has been solubilized (% g g^−1^)
