# Supplementary material for: Model-based optimization and scale-up of multi-feed simultaneous saccharification and co-fermentation of steam pre-treated lignocellulose enables high gravity ethanol production
Source: Biotechnol Biofuels. 2016 Apr 18;9:88. doi: 10.1186/s13068-016-0500-7 (PMC4835939; doi:10.1186/s13068-016-0500-7)
Supplement: Supplementary file 1 — 10.1186/s13068-016-0500-7 Contains experimental results from enzymatic hydrolysis experiments [file 13068_2016_500_MOESM1_ESM.pdf]

Model-based optimization and scale-up of multi-feed simultaneous saccharification and co-fermentation of steam-preatreated lignocellulose enables high gravity bioethanol production

Supplementary material

Table S1

Ruifei Wang [1], Pornkamol Unrean [1,2], Carl Johan Franzén[1§]

[1]Chalmers University of Technology, Department of Biology and Biological Engineering, Division of Industrial Biotechnology, Gothenburg, Sweden

[2]Current address: National Center for Genetic Engineering and Biotechnology (BIOTEC), Pathum Thani, Thailand

[§]Corresponding author: Carl Johan Franzén franzen@chalmers.se

Table S1. Summary of fed-batch enzymatic hydrolysis of steam pre-treated wheat straw.

Fed-batch hydrolysis [a]	No. of solid feeding events	Last feed at (h)	Final WIS (% w/w)	Enzyme feeding[b]	Glucose 96 h (g/L)	Glucose 120 h (g/L)	Yield[c] 96 h (%)	Yield[c] 120 h (%)
Equal solid feeding	6	72	15.1	Y	67.9	-	67.3	-
Equal solid feeding	6	72	15.1	N	81.9	-	82.0	-
Equal solid feeding	6	48	25.0	N	90.3	91.6	46.1	46.9
Exponential solid feeding	6	48	25.0	N	97.1	97.6	50.0	50.2
Model-based solid feeding	4	120	18.7	Y	74.1[d]	86.6[e]	61.9[d]	65.7[e]
Model-based solid feeding	4	120	18.7	N	85.5[d]	94.7[e]	71.7[d]	72.3[e]

[a]All fed-batch enzymatic hydrolysis started with 7% (w/w) WIS content.

[b]Y indicates enzymes were fed along with solid substrates, N indicates all enzymes were loaded at the start of experiments.

[c]The yields represent the % (w/w) of glucose released from added glucan.

[d]At 96h, the overall WIS reached 17.3% (w/w). The last solid feed according to modeling was conducted at 120h.

[e]Final sample was taken at 144 h.
