# Supplementary material for: Model-based optimization and scale-up of multi-feed simultaneous saccharification and co-fermentation of steam pre-treated lignocellulose enables high gravity ethanol production
Source: Biotechnol Biofuels. 2016 Apr 18;9:88. doi: 10.1186/s13068-016-0500-7 (PMC4835939; doi:10.1186/s13068-016-0500-7)
Supplement: Supplementary file 2 — 10.1186/s13068-016-0500-7 Contains experimental results from multi-feed SSCF experiments [file 13068_2016_500_MOESM2_ESM.pdf]

Model-based optimization and scale-up of multi-feed simultaneous saccharification and co-fermentation of steam-preatreated lignocellulose enables high gravity bioethanol production

Supplementary material

Table S2

Ruifei Wang [1], Pornkamol Unrean [1,2], Carl Johan Franzén[1§]

[1]Chalmers University of Technology, Department of Biology and Biological Engineering, Division of Industrial Biotechnology, Gothenburg, Sweden

[2]Current address: National Center for Genetic Engineering and Biotechnology (BIOTEC), Pathum Thani, Thailand

[§]Corresponding author: Carl Johan Franzén franzen@chalmers.se

Table S2. Summary of fed-batch multi-feed SSCF of steam pre-treated wheat straw [a].

Fed-batch SSCF[b]	No. of solid feeding events	Last feed at (h)	Final WIS (% w/w)	Enzyme/ Cell feeding[c]	EtOH 96 h (g/L)	EtOH 120 h (g/L)	Yield[d] 96 h (%)	Yield[d] 120 h (%)	Glucose 96 h (g/L)	Glucose 120 h (g/L)
Equal solid feeding every 12 hours	6	72	15.1	Y/N	44.2(2.8)	44.7	77.8(5.0)	78.7	0.08(0.03)	0.04
	6	72	15.1	Y/Y	48.2	50.0	84.9	88.0	0.2	3.5
	6	72	15.1	N/N	46.3(4.1)	46.0	81.5(3.6)	81.0	0.24(0.18)	0.22
	6	72	15.1	N/Y	48.8	52.3	86.0	92.0	0.1	0.6
Solid feeding every 6 hours in the day and 12 hours in nights	9	96	22.3	Y/N	33.9	32.7	36.3	35.2	48.6	55.3
	9	96	22.3	Y/Y	28.4	27.6	30.4	29.8	49.1	57.7
	9	96	22.3	N/N	29.1	28.6	31.1	30.8	59.2	65.8
	9	96	22.3	N/Y	42.1	41.8	45.0	45.0	38.2	42.7
Model-based solid feeding	6	96	22.2	N/N	43.1(6.5)	41.1(3.9)	49.4(7.1)	44.8(4.3)	26.0(4.6)	36.0(4.3)
	6	96	22.2	N/Y	57.3(1.1)	55.6(0.7)	65.6(1.3)	60.7(0.7)	4.7(1.5)	9.9(0.7)
Demo plant	5	72	21.8	N/Y	52.1(0.7)	-	54.4(0.07)	-	15.2(0.5)	-

[a]Results of duplicate experiments were reported as mean(SD) (n=2).

[b]All fed-batch SSCF started with 7% (w/w) WIS content.

[c]Y indicates enzymes or cells were fed along with solid substrates, N indicates all enzymes or cells were loaded at the start of experiments.

[d]The yields indicate % of the theoretical overall yield of ethanol on all sugar inputs to the bioreactor.
